# A Procedural Framework for Benchmarking Biofoundry Capabilities

**DOI:** 10.1021/acssynbio.3c00491

**Published:** 2023-11-09

**Authors:** Nathan J. Hillson

**Affiliations:** † United States Department of Energy Agile BioFoundry, Emeryville, California 94608, United States; ‡ Biological Systems and Engineering Division, Lawrence Berkeley National Laboratory, Berkeley, California 94720, United States

**Keywords:** biofoundry, benchmarking, business development, capability development

## Abstract

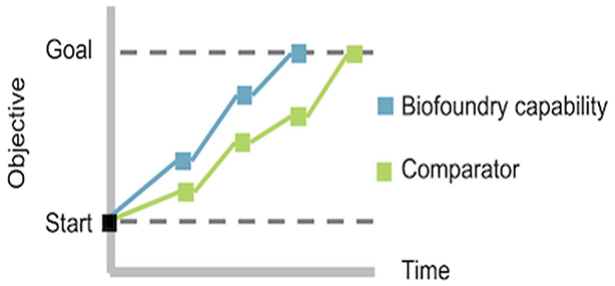

Benchmarking compares the performance of a product or service with a competitor. In a biofoundry context, capability benchmarking enables more effective use of development resources and furthering business development efforts. Biofoundries considering benchmarking activities are immediately faced with many implementation questions and decisions. While differing circumstances between biofoundries may lead to different answers to those same questions, a common framework for the benchmarking process is desirable. Perhaps the framework described here, and developed for the United States Department of Energy Agile BioFoundry, will be useful to other biofoundries around the world.

## Introduction

Benchmarking is the process of comparing the performance of a product or service against that of a competitor. Beyond showing how well a particular item rates in aggregate relative to its comparator, benchmarking can be used to identify specific underlying areas for improvement (e.g., a mobile phone has better battery life but is noticeably heavier than the competitor). This comparator can come from the same category (e.g., telephone vs telephone) or from another category (e.g., telephone vs telegraph) if the same objective can be accomplished across categories. For first-of-its-kind products/services/processes, benchmarking may not be appropriate, as one could simply not achieve the same objective otherwise (e.g., land on the moon).

In a biofoundry context, a capability is an ability to achieve a specific outcome or objective (e.g., design experiments, develop a microbial host, generate proteomics datasets in high throughput, run bioreactor fermentations, simulate processes, and analyze process techno-economics). While instruments, software, workflows, domain expertise, reagents, etc. underly capabilities, they are not capabilities per se. For example, a liquid-chromatography time-of-flight mass spectrometer is not itself a capability, but this instrument in conjunction with software, domain expertise, protocols, etc. constitute a biofoundry’s capability to quantify the metabolites in a sample. At times, the name of an instrument or software (etc.) can be used as a concise way to reference a closely associated capability that requires a lengthy description. For example, in the spreadsheet contained within the Supporting Information, the name of the software “Host Onboarding Tool (HObT)” is used in Column A to denote the capability to publicly share information about the status of microbial host development within the biofoundry, including associated publications, protocols, and strain and sequence information.

**Figure 1:**
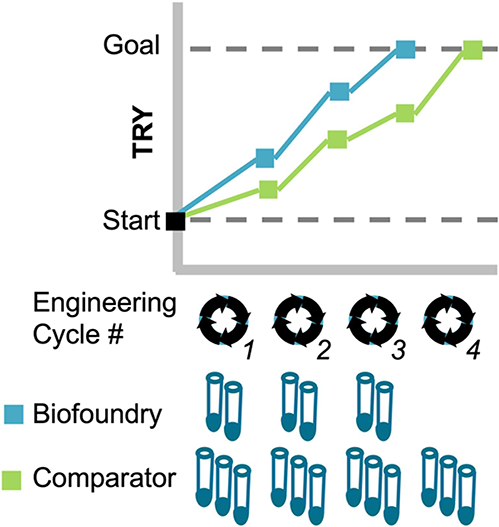
Representative benchmarking activity, comparing a biofoundry’s design of experiments capability with a leading method. For this example, the objective is to increase the titer, rate, and yield (TRY) of a microbial biochemical production process from a starting point to a prespecified goal and to do this as efficiently as possible (e.g., in the fewest cycles and with the fewest samples per cycle). The biofoundry capability is able to achieve the goal in only three engineering cycles and six sample variations, while the comparator requires four engineering cycles and 12 sample variations.

For a biofoundry, benchmarking a capability can support business development. Benchmarking can establish the value proposition for a capability in terms of what a capability does, its use cases, and how it is differentiated from its next best alternative (see Figure 1). This information can guide capability marketing efforts and help provide justification as to why the capability (and not the competitor) should be used in collaboration with the biofoundry or licensed out for use in a company. Benchmarking is also internally important to a biofoundry in informing stage-gating decisions (i.e., periodically making decisions about the resourcing of future work depending on progress made to date) regarding which capabilities should be sunset (i.e., divested, in terms of use and further development), so that resources can be redirected to higher return-on-investment capabilities.

Many considerations need to be made when planning a set of benchmarking activities. Which capabilities should be benchmarked? When should benchmarking be performed (during capability development or at maturity)? Which comparator and use case scenario should be evaluated? Who are the prospective customers? What is a sufficient demonstration for a prospective customer? How is performance measured? How to perform the benchmarking? How to determine when capability use and development should sunset or escalate? How should benchmarking results be disseminated? These questions are addressed through the process described below, which was recently developed for use by the U.S. Department of Energy Agile BioFoundry but could be of use to other biofoundries. For example, members of the Global Biofoundries Alliance,^1^ of which there are now more than 30 members (biofoundries.org), could effectively follow a similar process as a key part of building out their biofoundries.^2^

## Results and Methods

### Prioritize Capabilities for Benchmarking

#### Develop a List of Eligible Capabilities for Benchmarking

Start with the full list of the biofoundry’s capabilities. If that list does not exist or is not current, create it or update it. Exclude capabilities that are not usable yet and those that have been marked for divestment.

#### Categorize Primary Intention for Benchmarking

One reason for benchmarking is to focus future development efforts (where are the best opportunities to catch up, concede, and/or gain ground against a comparator?). Another reason for benchmarking is for stage-gating the development or maintenance process, with potential outcomes: immediately sunsetting, continuation as planned, ramping up, or restarting development. Yet another reason for benchmarking is to support business development efforts—to show why and how a capability should be used. It is important to choose the primary intention early in this process, as that choice greatly influences subsequent decisions (e.g., focusing future development efforts would have an internal audience, whereas supporting business development efforts would have an external audience). The benefits of benchmarking may not be exclusive to the primary intention (e.g., benchmarking to support business development can also inform future development and stage-gating decisions). Benchmarking should be coordinated with complementary approaches, such as conducting customer discovery interviews to better understand unmet needs to focus future development efforts.

#### Establish the Best Target Audience, Use Case, and Comparator for Each Capability

Benchmarking exercises are relative to a specific comparator and use case. When determining the best comparator and use case for a capability, there are several questions to be considered. Who is the target audience (internal to the biofoundry: development and leadership teams; external to the biofoundry: prospective collaborators and prospective licensees)? What comparator/use case would be the most appropriate for that target audience? How to ensure that the target audience agrees with the results? For any given capability there could be many target audiences, comparators, and use cases. Priority should be given to target audiences, comparators, and use cases that promise minimal costs and maximal benefits with clear differentiated outcomes from not having done the benchmarking.

#### Establish the Performance Criteria and Necessary Study Size for Each Capability

The qualitative criteria are set by the use case (e.g., detect a set of metabolites), but the precise quantitative targets (e.g., same or better detection limit as the comparator but with a 50% reduction in method time) have yet to be set. The quantitative criteria must be chosen so as to convince a majority of the target audience. For business development, what is a sufficient demonstration (scale, performance level, reproducibility, versatility, absolute or relative to comparator, statistical significance) for the prospective customer to choose the capability? For stage-gating, what is a sufficient demonstration for the biofoundry leadership to continue (or increase) resourcing the development or maintenance of the capability? For focusing development efforts, what is a sufficient demonstration to inform the development team about the best next steps of the capability to invest in? The necessary study size will generally be set by the performance criteria (e.g., enough samples to show reproducibility, versatility, statistical significance). However, study size may need to be expanded to provide a solid and clear signal for next steps (e.g., stage-gating or path forward for licensing).

#### Estimate the Benchmarking Costs for Each Capability

A back of the envelope calculation is sufficient, as the actual benchmarking work has yet to be fully designed. It is anticipated that there will be significant error bars in these cost estimates, which should be captured as possible and considered while making prioritization decisions. To the extent possible, benchmarking experiments should direct work in ongoing biofoundry projects without significant changes to workflow (e.g., just different choices of media formulation), to minimize counterfactual costs—those above and beyond what would already be incurred for the ongoing project (e.g., additional work needed specifically for benchmarking). However, at times, more dramatic changes will need to be made (e.g., study size needs to be increased to achieve needed statistical significance or samples need to be prepared differently for a different instrument). There may also be costs associated with licensing (e.g., commercial software) and training biofoundry staff on the use of the identified comparator. Finally, there will be costs associated with designing the experiments and analyzing the resulting data.

#### Estimate the Benchmarking Benefits for Each Capability

This estimate should also be counterfactual and back of the envelope, with error bars captured and considered as above for the cost estimate. There are several different types of benefits that could result from benchmarking, such as freeing up resources (should a capability be sunset), better use of resources (with more focused development), establishing more collaboration projects, and increased licensing. The extent to which biofoundry resources would be freed up by sunsetting, more quickly consumed by a ramp-up, or more effectively used through better-focused development, is directly calculable by the biofoundry. The prospective benefits through additional collaboration projects or licensing should be ascertained through discussions with prospective customers. What may still need to be estimated is the total number of prospective customers that are covered by the same target audience archetype. Each benchmarking activity will likely have a variety of possible outcomes. For example, a capability status could be changed to active development, maintenance, or sunsetting depending on the results (and on the stage-gating criteria), or the scale of a collaboration project could grow substantially as a function of how much better the biofoundry capability is than the comparator. Since there will be multiple possible outcomes for each benchmarking activity, each outcome should be weighted by its estimated probability of occurrence.

#### Rank Capabilities Given Estimated Costs and Benefits

**Figure 2:**
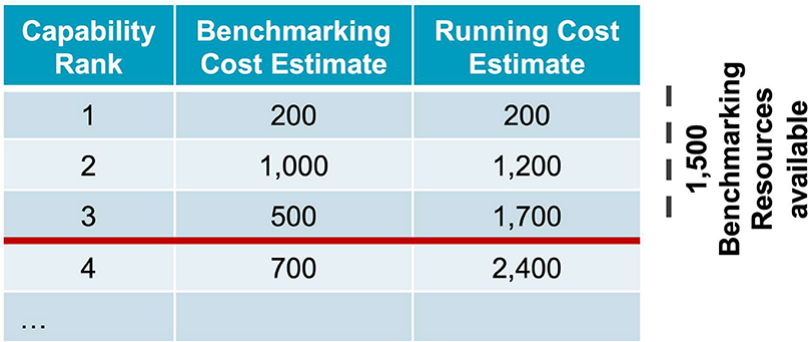
Example of determining which capabilities progress into benchmarking design. Capabilities are ranked by return on investment (counterfactual weighted average benefit divided by cost). The running cost estimate (1,700) for the three top-ranking (highest priority) capabilities crosses the amount of resources available for benchmarking (1,500), so only these three capabilities will progress into benchmarking design.

The rank ordering should be performed considering return on investment (counterfactual weighted average benefit divided by cost). With the rank ordering completed, compute a running estimated cost beginning with the highest-ranked (top priority) capability and adding the cost for each following (by rank) capability progressively. The highest-ranked capabilities, up until the running cost estimate crosses the amount of resources available for benchmarking, will progress into benchmarking design (see Figure 2). The main risk to advancing too many capabilities (e.g., more than the amount of resources available) into benchmarking design is that resources will be insufficient to do all of the designed benchmarking.

#### Use Spreadsheet to Facilitate Prioritization of Capabilities for Benchmarking

The Supporting Information consists of a spreadsheet used to facilitate our prioritization process. Example content is provided in Row 2 of the spreadsheet for a capability concerning publicly sharing information about the status of microbial host development within our biofoundry, including associated publications, protocols, and strain and sequence information. Such example content should be helpful to other biofoundries preparing analogous content for their capabilities as they prioritize their own capabilities for benchmarking.

### Design Prioritized Benchmarking Activities

The benchmarking activities were sketched at a high level above, and specific details need to be set in terms of timing (e.g., coordination with ongoing biofoundry work), specific use cases, specific sample specifications (e.g., media formulations, genetic modifications, etc.), comparator configurations, etc. Once the full specification details have solidified, it is important to recheck the refined design in terms of continuing to satisfy the target audience and of being relevant to the intended use case and comparator. For business development purposes, where possible, it is desirable to approach the target audience with the plan to reconfirm interest and the extent of demonstration sufficiency. This is an opportunity to make any needed adjustments to the designed specification details.

As the design becomes finalized, it will be possible to more precisely and accurately estimate the counterfactual costs and benefits. In cases where estimated counterfactual costs or benefits have changed significantly, biofoundry leadership will likely need to reapprove the benchmarking activity before proceeding. If not approved, the capability would move down in the benchmarking prioritization queue. It is very important at this stage (just before the actual benchmarking activity begins) for the biofoundry leadership, in light of the finalized benchmarking design, to review and reconfirm the stage-gating criteria.

### Benchmark

The benchmarking work should be initiated with a meeting in which expectations for the capability developers and for the biofoundry project collaborators are clearly laid out. These expectations need to be understood and agreed to by all contributors to the designed benchmarking work plan. This initial meeting should be led by the benchmarking activity designers, who should ensure that all essential information is effectively disseminated. At this time, it may be necessary to procure the comparator (e.g., software, dataset, instrument, etc.) as needed.

Science and technology development can change directions quickly. If a biofoundry project were to be significantly modified or terminated in the middle of a benchmarking activity, an assessment needs to be made (a joint effort between the benchmarking team and biofoundry leadership) to decide if continuing the project for the benefit of the benchmarking activity is justified or if benchmarking should be redesigned or deprioritized.

### Assess Results, Make Stage-Gate Decisions, and Implement and Monitor Changes

After benchmarking has completed, the next step is to assess the results against the comparator and the statistical significance of that comparison and, based on the stage-gating criteria, determine whether the capability status should be changed (i.e., to increased active development, maintenance, or sunsetting) and obtain biofoundry leadership concordance with this decision. In instances where benchmarking informs the prioritization of development within a capability, this is also the time to make these assessments.

The results of benchmarking activity should be reported to the biofoundry leadership. This reporting should include the assessment made by the benchmarking team as to which stage-gate decision is supported by the results, along with any notes for consideration (either supporting, providing reservations, or suggesting alternative outcomes) when the biofoundry leadership is evaluating the stage-gate decision. The biofoundry leadership then evaluates the benchmarking results summary and notes and makes a decision on the stage-gating outcome for each benchmarked capability. The biofoundry leadership then works with the capability development team to propose a change of course (if needed) following the decision and periodically check that the changes are being implemented as planned. Note that the biofoundry use of a capability and the development of the capability are related, with the usage arc lagging behind development. That is to say, when development of a capability has sunset (i.e., it is no longer being maintained), there may be a short period when use of the capability continues, but then usage too must be sunset (once lack of capability maintenance has eliminated its functionality).

### Disseminate Results

Dissemination to internal and external audiences can take place in a variety of forms, either as information becomes available or as rolled into periodic reporting documentation. At the conclusion of each benchmarking activity, a synopsis of the benchmarking should be appended to an internal biofoundry benchmarking report, including the target audience, use case, comparator, and study size; stage-gating metrics for active development, maintenance, and sunsetting; summarized results; and the decision made regarding the capability status (e.g., to sunset). The conclusion of the benchmarking activity is also a good time to capture the actual counterfactual costs incurred by the benchmarking, in comparison to what was estimated, and document this difference, so as to learn from this experience to improve future benchmarking cost estimations.

At the conclusion of each benchmarking activity, several more externally facing disseminations should be made. For benchmarked capabilities showing positive performance (against the comparator) that have yet to be included on the biofoundry website, the capability description needs to be added. For capabilities already on the biofoundry website, descriptions should be updated as appropriate. For capabilities that have been sunset, their descriptions could be moved to a section of the biofoundry website dedicated to “previous work” (or similar). Business development materials, akin to the biofoundry website, should be updated to feature or delist these same capabilities. Regarding business development, the target audience of the benchmarking should be directly contacted with the results. Where appropriate, the biofoundry should seek a peer-reviewed publication for the benchmarking activity and generate a summary report for an external audience. Once available, these publications and reports should be promoted via the biofoundry’s social media and email lists, with links to the capability descriptions on the biofoundry website.

On a periodic basis, it is important to further document benchmarking activities, results, and outcomes. This includes generating (or updating the previous version, if available) a table of the steps above, as an at-a-glance reference, as well as an appendix that shows the work and evidence behind what is shown in the summary table. It is important to include in this documentation newly accumulated learnings regarding comparisons of estimated counterfactual benefits with actual benefits realized and similarly the actual behaviors of target audiences following benchmarking results compared with how they said they would behave. This documentation should be made internally accessible to biofoundry contributors as well as to the entities supporting the biofoundry.

## Discussion

**Figure 3:**
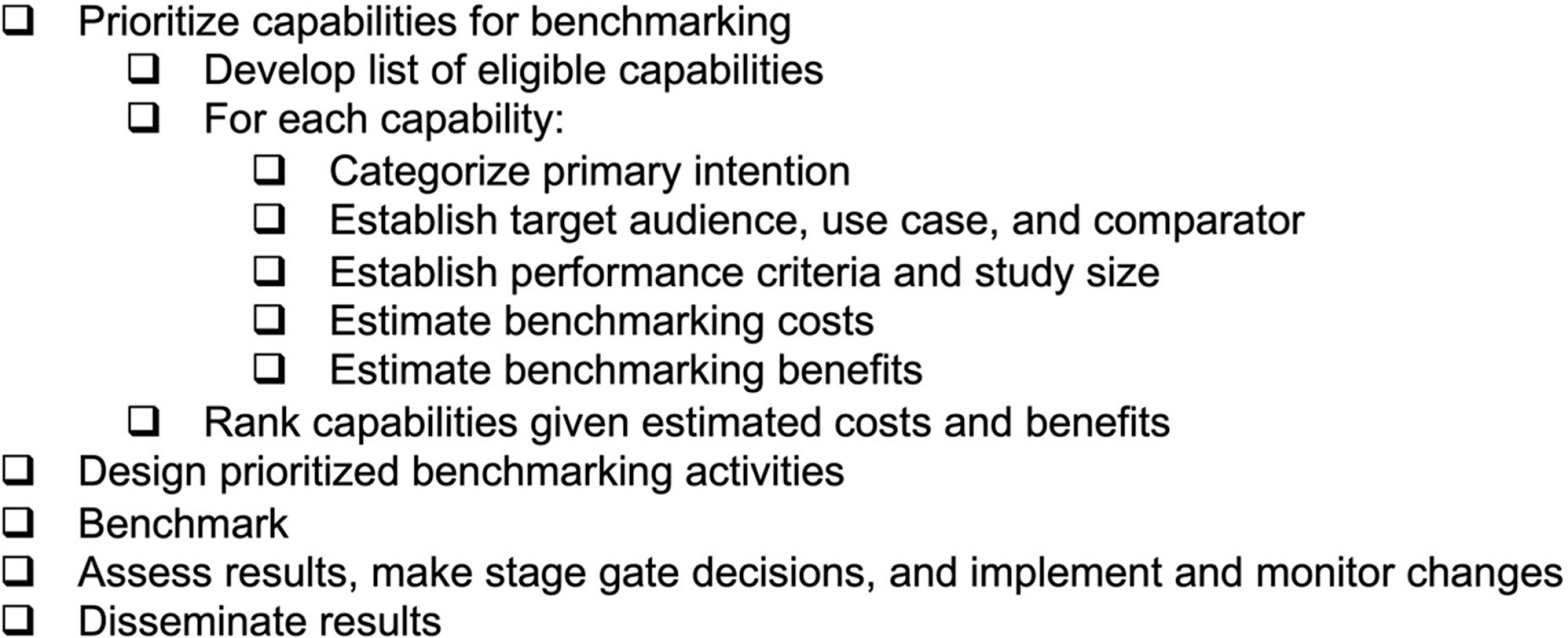
Checklist for the described biofoundry capability benchmarking process.

In a biofoundry context, capability benchmarking can be an effective approach for furthering business development efforts and making more efficient use of development resources. While the general concept of benchmarking is simple to describe, there are many questions and decisions that need to be made when going about it in practice. The process described above (and summarized as a checklist in Figure 3), developed for use within the Agile BioFoundry, is but one possible implementation. Perhaps the above framework will be helpful to other biofoundries as they pursue their own capability benchmarking activities.

## Supporting Information Available

“Benchmarking prioritization example.xlsx” – Microsoft Excel format spreadsheet file. Row 2: Example content for a representative capability going through the benchmarking prioritization process. Note: Columns U (“ROI Rank”) and V (“Running benchmarking cost”) have placeholder values, as specific numbers would only apply in the context of other listed capabilities (XLSX)

## Abbreviations

TRY: titer, rate, and yield
